# Executive Functions and Emotional Granularity: No Evidence for Positive Associations

**DOI:** 10.1007/s42761-025-00307-y

**Published:** 2025-05-23

**Authors:** Marcel C. Schmitt, Julia Karbach, Tanja Könen, Ulrike Basten, Julia A. Glombiewski, Tina In-Albon, Tanja Lischetzke

**Affiliations:** 1 RPTU University Kaiserslautern-Landau, Fortstr. 7, 76829 Landau, Germany; 2 University of Mannheim, Mannheim, Germany

**Keywords:** Emotional Granularity, Emotion Differentiation, Executive Functions, Emotion Categorization, Chronic Pain

## Abstract

Emotional granularity is the ability to experience one’s emotional experiences in a nuanced and specific manner. According to the theory of constructed emotion, between-person differences in emotional granularity are a function of how efficiently individuals categorize affect-related input by means of emotion concepts. We propose that executive functions support the effective coordination of the emotion categorization process, thereby facilitating emotional granularity. In the present study, we empirically examined the associations between three domains of executive functions (i.e., working memory, inhibition, and shifting) and emotional granularity for both negative and positive emotions (negative and positive emotional granularity) in two samples (participants with and without chronic pain). We hypothesized that all three executive function domains would be positively associated with both negative and positive emotional granularity. Participants (*N* = 153 individuals without chronic pain, *N* = 218 individuals with chronic pain) completed an online assessment of executive functions and a 14-day ambulatory assessment of 15 negative and 12 positive emotions (with five prompts per day). Contrary to our expectations, neither the six manifest executive function task scores nor a latent common executive function factor measured by the task scores were significantly positively associated with the negative and positive emotional granularity indices in either sample and in analyses that combined both samples. Our findings suggest that executive functions may play a lesser role for emotional granularity than previously assumed. We discuss alternative operationalizations of executive functions and emotional granularity that might reveal stronger associations between these constructs.

**Supplementary Information:**

The online version contains supplementary material available at 10.1007/s42761-025-00307-y.

*Emotional granularity* or emotion differentiation is the ability to generate nuanced and differentiated emotional experiences and distinguish between like-valenced emotional states (Barrett et al., 2001; Kashdan et al., 2015; Seah & Coifman, 2022). Individuals with high granularity tend to experience distinct emotions in a context-specific manner (e.g., feeling sad, but not angry), whereas individuals with low granularity tend to have more diffuse affective emotional experiences (e.g., feeling bad). High emotional granularity has been associated with various positive outcomes in non-clinical and clinical populations, such as higher emotion regulation success (Kalokerinos et al., 2019) or lower levels of depressive symptoms (Starr et al., 2017; for meta-analyses and reviews, see O’Toole et al., 2020; Seah & Coifman, 2022; Thompson et al., 2021).

Given the importance of emotional granularity for psychological functioning, understanding the mechanisms behind individual differences in emotional granularity is crucial. An important framework for the mechanisms of emotional granularity is the theory of constructed emotion (formerly known as conceptual act theory of emotion; Barrett, 2014, 2017a, 2017b). This theory proposes that emotions are predictive constructions by the human brain to make sense of an individual’s affective state in a given situation (Barrett, 2017a, 2017b). When individuals experience a particular emotion, they categorize that affective state as an emotional instance by drawing on emotion concepts, which are dynamic mental representations of emotion categories based on past experiences (Barrett, 2006). For instance, someone experiencing anger in a particular situation uses the concept of anger, considering this concept to be the most appropriate representation of his or her affective state (Lee et al., 2017).

The process of categorizing emotions is intricate and involves two interdependent cognitive subprocesses: first, the processing of affect-related input from the body (e.g., high heart rate) and the environment (e.g., blocked goals), and second, the application of emotion concepts that best match the input based on individuals’ past experiences (Lee et al., 2017; Lindquist & Barrett, 2008). High emotional granularity depends on the efficient coordination of these subprocesses, allowing individuals to draw on context-specific emotion concepts rather than relying on broader categorizations that result in generalized feelings of (un)pleasantness (Lee et al., 2017; Lindquist & Barrett, 2008). Thus, the ability to orchestrate the integration of affect-related input and the use of emotion concepts in the emotion categorization process should be key to high emotional granularity.

Coordinating the two cognitive subprocesses of emotion categorization likely involves executive functions—higher-order cognitive control processes that “regulate the dynamics of human cognition and action” (Miyake & Friedman, 2012, p. 8). Indeed, individual differences in executive functions have been mentioned as potential antecedents of emotional granularity in the literature (Kashdan et al., 2015; Lee et al., 2017; Lindquist & Barrett, 2008), but empirical underpinnings of this proposed link are limited (Lee et al., 2017). Addressing this gap, the present study empirically examined the association between executive functions and emotional granularity.

## Domains of Executive Functions and Emotional Granularity

It has been proposed that executive functions include three moderately correlated core domains (Miyake et al., 2000): *working memory*, *inhibition*, and *shifting*. Working memory describes the ability to hold information in mind while simultaneously manipulating it. Inhibition refers to the ability to suppress irrelevant information and prepotent impulses to act. Finally, shifting is described as the ability to flexibly switch between different response rules, perspectives, or mental tasks (Diamond, 2013).

We propose that each of the three domains serves a different function in coordinating the emotion categorization process. First, a high working memory capacity should allow individuals to maintain and integrate a large amount of diverse affect-related information from the body and environment (e.g., arousal, contextual information) while simultaneously retrieving their emotion concept knowledge. The more contextual information is available and actively maintained, the better individuals can select precise emotion concepts that best fit the specific situation (Lee et al., 2017). Second, individuals with high inhibitory control should be able to suppress more readily available but imprecise emotion concepts when other emotion concepts are more appropriate for the current affective state (Lee et al., 2017; Lukic et al., 2023). Finally, shifting should allow individuals to quickly revise the emotion categorization process when new relevant affective information becomes available in a given situation, allowing them to switch to more appropriate emotion concepts as the situation requires it.

Indirect support for a link between executive functions and emotional granularity comes from two neurophysiological studies: One study, which used electroencephalography while participants processed emotional stimuli, found that participants with higher emotional granularity had a more negative amplitude in the N2, a component of event-related potentials that is associated with executive control (Lee et al., 2017). In a recent neuroimaging study, higher emotional granularity was associated with greater cortical thickness of the lateral orbitofrontal cortex, a brain region associated with the controlled selection of semantic concepts and behavioral inhibition (Lukic et al., 2023). However, establishing a direct link between executive functions and emotional granularity requires assessing executive functions with cognitive tests rather than relying solely on neural correlates.

The only study to empirically investigate a direct link between executive functions and emotional granularity through cognitive assessments was an unpublished study by Barrett and colleagues (cited in Lee et al., 2017). The authors observed a positive correlation between higher working memory capacity, as measured by several complex span tasks, and granularity of negative emotions (negative emotional granularity). While this unpublished study provides preliminary evidence for a positive association between working memory and negative emotional granularity, it did not examine granularity of positive emotions (positive emotional granularity) and the other two executive function domains.

To our knowledge, no study has examined how all executive function domains relate separately to both negative and positive emotional granularity. The present study combined online executive function tasks assessing all three executive function domains with a 14-day ambulatory assessment of participants’ negative and positive emotions, using data from a collaborative research project investigating emotional processes in individuals without and with chronic pain. As preregistered, our primary focus was on individuals without chronic pain. However, to assess the robustness and generalizability of our findings, we also analyzed data from the chronic pain sample and additionally combined the two samples to increase statistical power, without expecting differences in the mechanisms linking executive functions and emotional granularity between the two groups. We hypothesized that the executive function domains under study (working memory, inhibition, and shifting) would be positively associated with both negative and positive emotional granularity. We preregistered these hypotheses and our analysis plans prior to data collection (https://osf.io/usvcz/).

## Method

### Procedure

Participants were recruited throughout Germany through various channels, including newspaper ads, social media and flyers in hospitals and doctor’s offices. Upon registration, participants completed an online screening questionnaire, reporting demographics and regular pain experiences. Participants were eligible if they were at least 14 years old (with parental consent for participants under 16) and had access to a smartphone and a home computer. Participants were eligible if they were at least 14 years old (with parental consent for participants under 16) and had access to a smartphone and a home computer.

The study consisted of an online trait survey, online executive function tasks, a 14-day ambulatory assessment of emotional experiences, and laboratory emotion regulation tasks. Only the parts relevant to the present paper are described. In the online executive function assessment, participants completed six tasks, divided into two sessions (approximately 40–45 min), on their home computers on two separate days. Each session contained three different tasks, with each task designed to tap one of the three executive function domains. The order of the executive function domains assessed by the tasks in each block was counterbalanced across sessions.

For the subsequent 14-day ambulatory assessment, participants were asked to install SEMA3 (O’Brien et al., 2024) or m-Path (Mestdagh et al., 2023) on their smartphones, depending on the compatibility of their operating systems. Participants could choose between two time schedules (8 am to 9 pm or 9 am to 10 pm on weekdays with a shift an hour later on weekends) that best fit their usual waking hours. Participants received five prompts per day to take part in a short survey according to a stratified random interval scheme. Each prompt was active on the smartphone for 30 min. If participants did not respond to the initial beep of a prompt, they received a reminder 15 min after the initial beep. The short surveys consisted of 61 to 79 items, depending on the time of the day and a filter question not relevant to the present study, which asked participants, among others, about their recent emotional experiences. The average time participants spent on a survey was 4.35 min.

Data collection took place from April 2022 to March 2024. Participants received financial reimbursement of up to €150. The study was approved by the local ethics committee (application LEK-397).

### Participants

A total of 644 German-speaking individuals enrolled in the overall study and completed the initial online screening questionnaire. Of these, 375 were classified as having chronic pain, as they reported experiencing regular pain for at least 3 months (according to the classification of chronic pain in the ICD-11; Treede et al., 2015) in this questionnaire. In the following, we will refer to the subsample of participants without chronic pain as Sample 1 and those with chronic pain as Sample 2. Of all 644 registered participants, 495 participants (211 in Sample 1, 284 in Sample 2) completed the online executive function tasks, and 418 participants (176 in Sample 1, 242 in Sample 2) enrolled for the subsequent ambulatory assessment. Our final analyses were based on *N* = 153 participants without chronic pain (Sample 1; age: *M* = 39.61 years, *SD* = 15.52, *Min* = 14, *Max* = 78; gender: 43% male, 56% female, 1% non-binary) and *N* = 218 participants with chronic pain (Sample 2; age: *M* = 39.67 years, *SD* = 15.92, *Min* = 14, *Max* = 83; gender: 26% male, 70% female, 4% non-binary) who completed the online executive function tasks and provided valid data on at least 30% (i.e., 21) of all occasions in the ambulatory assessment phase, totaling 8,113 and 11,662 occasions in Samples 1 and 2, respectively (see the section Data cleaning for details).

The samples varied regarding the highest school qualifications (Sample 1: 13% lower secondary school diploma, 30% higher secondary school diploma, 20% bachelor’s degree, 30% master’s degree, and 4% PhD; Sample 2: 26% lower secondary school diploma, 32% higher secondary school diploma, 20% bachelor’s degree, 20% master’s degree, and 1% PhD). Psychological disorders were more prevalent in Sample 2 (29%, *n* = 63) than in Sample 1 (5%, *n* = 8). Specifically, Sample 1 included 3% with a depressive disorder, 2% with a bipolar disorder, 1% with an attention deficit disorder, and 1% with a borderline personality disorder. In Sample 2, 17% reported a depressive disorder, 10% an anxiety disorder, 7% posttraumatic stress disorder, 2% attention deficit disorder, 1% obsessive–compulsive disorder, and 6% reported other diagnoses. Multiple diagnoses were common in this sample.

### Materials

#### Executive Function Tasks

The six executive function tasks were computerized adaptations of established paradigms. We used accuracy scores (i.e., percentages of correct responses), rather than response time measures, to benefit from enhanced reliability (Draheim et al., 2021). Working memory was assessed by a Visual-Verbal Complex Span Task (VCST; Kane et al., 2004) and a Spatial *n*-Back Task (SNT; Schmiedek et al., 2010); inhibition by an Antisaccade Task (AST; Kane et al., 2001) and the Sustained Attention-to-Cue Task (SACT; Draheim et al., 2021); and shifting by the Wisconsin Card Sorting Test (WCST; Grant & Berg, 1948) and the Flexible Item Selection Task (FIST; Dick, 2014).

##### Visual-Verbal Complex Span Task (VCST; Working Memory)

Each trial consisted of an encoding phase and a recall phase. In the encoding phase, pictures of animals (horse, pig, dog, or cow) were presented upright or upside down, one at a time. Participants were asked to indicate the direction of the animals by clicking on the left or right mouse button for each picture while memorizing the order in which the animals were presented. In the recall phase, all four animals were presented upright and participants were asked to recall the order of the presentation by clicking on the pictures sequentially in the order in which they were presented. The task consisted of two blocks of nine trials each. The trial load (i.e., the length of the animal series to be memorized) increased after every three trials, starting with a load of two and reaching a maximum load of seven. The overall percent correct score included correct responses from both the encoding and recall phase. Spearman-Brown corrected split-half reliabilities, calculated using an even–odd split of trials, were .92 in Sample 1 and .91 in Sample 2 for this score.

##### Spatial *n*-Back Task (SNT; Working Memory)

A series of stimuli in the form of flowers appeared at different locations in a 4 × 4 matrix. Participants were asked to press the space bar on their keyboard if a stimulus appeared at the same location in the matrix as *n* steps earlier in the sequence, with two and three steps for the first and second phases, respectively. The 2-back phase consisted of two blocks with 38 trials each, whereas the 3-back phase consisted of two blocks with 39 trials each. In both phases, the order of the stimuli was randomized with the following restrictions: (1) Twelve items were targets (i.e., items that matched the stimulus *n* steps earlier). (2) Stimuli did not appear at the same location twice in consecutive trials. (3) In the 2-back phase, exactly three items each appeared at the same location as items three, four, five, or six steps earlier. In the 3-back phase, exactly three items each appeared at the same location as items four, five, or six steps earlier. (4) There were no lures with longer lags than six. Participants’ responses were measured as hits (correctly identified targets), omissions (missed targets), correct rejections (accurate non-target identification), and false alarms (incorrect non-target responses). The overall percent correct score included both hits and correct rejections. Spearman-Brown corrected split-half reliabilities were .90 in Sample 1 and .85 in Sample 2 for this score. For supplementary analyses, we calculated the percentages of omissions and false alarm as specific error scores.

##### Antisaccade Task (AST; Inhibition)

In each trial, a cue flashed (“ = ”) on either the right or the left side of the screen for 300 ms. After the cue disappeared, the target (either “B”, “P”, or “R”) appeared on the opposite side of the previous flashing cue for 150 ms. Participants were asked to identify the target by pressing the “7”, “8”, or “9” keys on their keyboard for the “B”, “P”, or “R” targets, respectively. The task consisted of three blocks with 24 trials each. Spearman-Brown corrected split-half reliabilities were .95 in each sample for the overall percent correct score.

##### Sustained Attention-to-Cue Task (SACT; Inhibition)

At the beginning of each trial, a cue in the form of a shrinking circle appeared at a random location on the screen. The cue was followed by a flashing distractor (“*”) in the center of the screen for 300 ms. A 3 × 3 letter matrix was presented at the location of the previous cue. The central letter of the matrix rep-resented the target (“B”, “D”, “P”, or “R”) and was replaced by a “#” after 125 ms. The masked matrix then disappeared and the participants were asked to indicate the central letter of the previous matrix by clicking on the corresponding box. The task consisted of two blocks with 32 trials each. Spearman-Brown corrected split-half reliabilities were .94 in Sample 1 and .96 in Sample 2 for the overall percent correct score.

##### Wisconsin Card Sorting Task (WCST; Shifting)

In all trials, four key cards were presented at the top of the screen. Each key card represented a different combination of three categorization criteria (symbol depicted: triangle, star, cross, circle; number of symbols: 1–4; color of symbol: red, green, yellow, blue). One response card at a time was presented at the bottom of the screen. Participants were asked to match the response card with one of the key cards regarding the current categorization rule (i.e., sorting by symbol, number, or color), which they had to find by trial and error by clicking on the corresponding key card. Participants received feedback on whether or not their categorization was correct. After ten correct categorizations, the categorization rule changed. The task was terminated after participants completed six sets of rules or after a maximum of 128 trials. The order of the categorization rules was the same across participants. Spearman-Brown corrected split-half reliabilities were .94 in each sample for the overall percent correct score. Although we preregistered using the number of perseveration errors (i.e., categorizations according to the previous rule after the categorization rule has changed), we decided on using the overall percent correct score for our main analyses, as the latter was less skewed and more consistent with the scores of the other tasks. In supplementary analyses, however, we used the percentage of perseveration errors along with the percentage of drift errors (i.e., false categorizations after having already responded correctly in a rule block) as specific error scores.

##### Flexible Item Selection Task (FIST; Shifting)

In each trial, participants were presented four cards that differed in the combination of four matching categories: picture (ship, rabbit, or rose), size of the pictures (large, medium, or small), number of the pictures (1–3), and color of the pictures (green, red, or blue). Participants were asked to find four different pairs of cards by clicking on the cards that belonged in a pair. Importantly, the matching categories had to differ across the four pairs. After a pair was selected, participants had to indicate the category according to which the pair matched by clicking on the box with the corresponding category. Participants received immediate feedback indicating whether their answer was correct and subsequently proceeded with the remaining pairs. The task consisted of 12 trials, each of which had to be completed within 14 s. Deviating from the preregistration, we did not use the percentage of correct responses for the fourth-pair selections as the FIST score, as many participants failed to reach this stage in time, leading to excessive missing data. Instead, we used the percentage of correct responses across all pair selections. Spearman-Brown corrected split-half reliabilities were .96 in Sample 1 and .95 in Sample 2 for this score.

#### Emotion Ratings

In each ambulatory assessment survey, participants were asked to indicate the highest intensity with which they had experienced 15 negative and 12 positive emotions in the past two hours (or, since getting up at the first prompt of the day) on 101-point slider scales ranging from 0 (*not at all*) to 100 (*very strongly*). The 27 emotion items were adapted from the modified Differential Emotions Scale (mDES; Fredrickson, 2013) and presented in a randomized order. As negative emotion items, we selected *angry*, *irritated*, *annoyed*, *scared*, *fearful*, *worried*, *sad*, *downhearted*, *unhappy*, *ashamed*, *humiliated*, *disgraced*, *hopeless*, *pessimistic*, and *discouraged*. As positive emotion items, we selected *joyful*, *glad*, *happy*, *interested*, *alert*, *curious*, *love*, *closeness*, *trust*, *serene*, *content*, and *peaceful*. Consistent with previous studies using slider scales (e.g., Koval et al., 2015; Lischetzke et al., 2021), we recoded all ratings ≤ 5 to 0 because it may have been difficult for participants to select a value of exactly 0 on smartphone touchscreens.

##### Emotional Granularity Indices

To obtain negative and positive emotional granularity indices, we computed person-specific intraclass correlation coefficients (ICC[3,*k*]) between emotion ratings across all measurement occasions, separately for negative and positive emotions (e.g., Lischetzke et al., 2021). The ICC theoretically ranges from 0 to 1, with a high ICC reflecting a consistent (non-)endorsement of different emotion items across occasions, indicating that the individual did not experience these emotions in a discrete or differentiated manner. While negative, and thus noninterpretable, ICC values have sometimes occurred for a small number of participants in previous studies, necessitating the exclusion of those values (e.g., Erbas et al., 2019), this issue did not arise in the present study. We subsequently Fisher *Z*-transformed and inverted the ICCs, such that higher values corresponded to higher emotional granularity.

##### Mean Levels of Negative and Positive Emotions

As control variables for supplementary analyses, we calculated mean scores for negative and positive emotions for each participant by first averaging across emotion items for each valence separately and then across all measurement occasions.

### Data Cleaning

For the preprocessing of the executive function data, we performed several data cleaning steps at the trial and task level before calculating the executive function task scores. First, across all tasks, trials with response times faster than 200 ms were excluded, as such low response times may indicate careless responding. Second, trials with response times that were 2.5 standard deviations below or above each participant’s mean response time for each task were excluded, as these were considered outliers. Third, we excluded scores of tasks in which participants encountered technical failures, as well as task scores that fell below the chance level (e.g., 33% for the AST), to ensure meaningful engagement with the tasks.

Of the 418 participants enrolling for the ambulatory assessment, 167 participants without (Sample 1) and 234 participants with chronic pain (Sample 2) provided data on at least one survey, for a total of 8,663 (Sample 1) and 12,502 (Sample 2) surveys. We performed several cleaning steps at the survey level: First, we removed 40 (Sample 1) and 39 (Sample 2) surveys with technical errors (e.g., prompts that were issued less than 120 min after the previous prompt). Second, we removed 24 (Sample 1) and 55 (Sample 2) surveys in which emotion items were unanswered. Finally, we removed surveys that were flagged as indicating careless responding in terms of response time and inconsistent response behavior (Meade & Craig, 2012). If surveys had response times below a certain cutoff, they were considered to be completed too quickly to be meaningful responses. Cutoffs for extremely fast response times averaged per item were determined in a pilot study in which research assistants were instructed to complete the surveys as quickly as possible without switching to careless responding for each of the two software programs separately. Inconsistent responding was determined by inspecting response patterns across two reverse-poled momentary mood items (i.e., ratings of < 20 or > 80 for both reverse-poled items were considered careless responding). In total, we removed 195 (Sample 1) and 363 (Sample 2) occasions due to careless responding. At the participant level, we only included data from participants who provided at least 21 (= 30%) valid (i.e., complete, non-careless) surveys to ensure that the emotional granularity indices were reliably calculated. The final Sample 1 (i.e., participants without chronic pain) consisted of 153 participants who completed an average of 53.03 surveys (76% of all surveys, *SD* = 12.07). They provided the following numbers of task scores: 148 for VCST, 150 for SNT, 143 for AST, 147 for SACT, 145 for WCST, and 151 for FIST. The final Sample 2 (i.e., participants with chronic pain) consisted of 218 participants who completed an average of 53.50 surveys (76% of all surveys, *SD* = 11.13). They provided the following numbers of task scores: 215 for VCST, 214 for SNT, 206 for AST, 207 for SACT, 215 for WCST, and 218 for FIST.

### Statistical Analyses

**Fig. 1:**
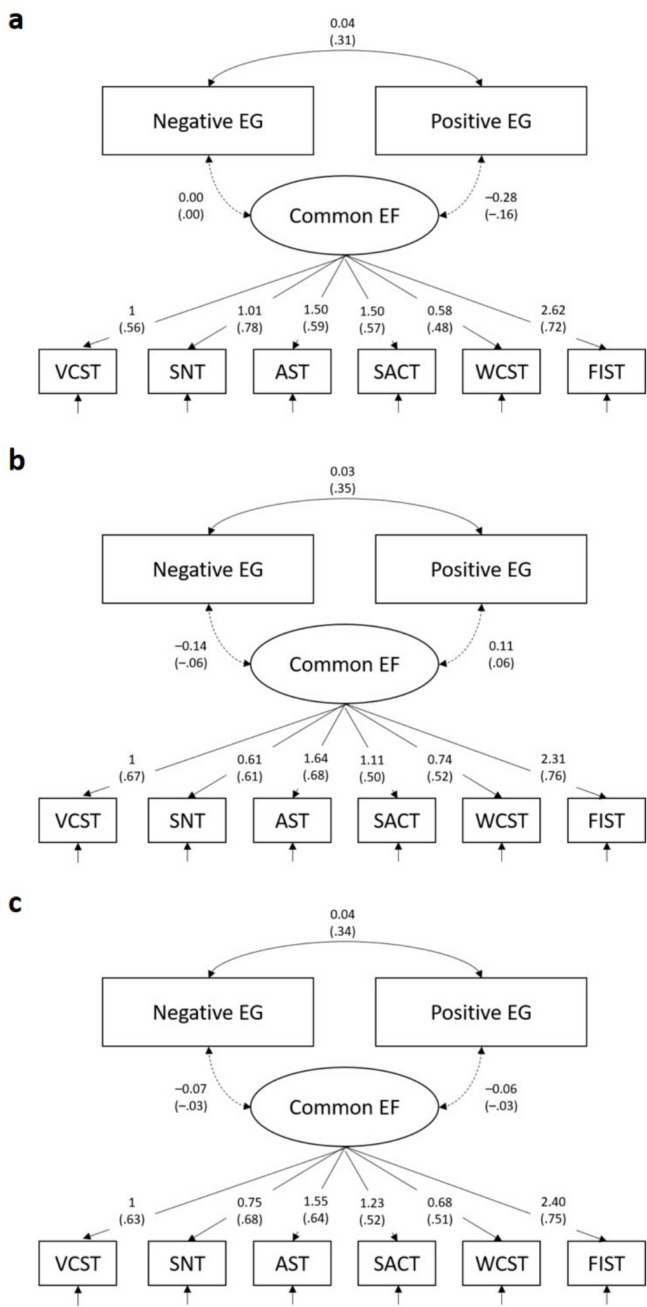
Measurement model for the common executive function factor and correlations with negative and positive emotional granularity indices in (**a**) Sample 1, (**b**) Sample 2, and (**c**) in the combined sample. Values without parentheses indicate unstandardized values (i.e., unstandardized factor loadings, covariances), values in parentheses indicate standardized values (i.e., standardized factor loadings, correlations). Dashed arrows represent correlations not significantly greater than zero. EG = emotional granularity; EF = executive functions; VCST = Visual-Verbal Complex Span Task; SNT = Spatial n-Back Task; AST = Antisaccade Task; SACT = Sustained Attention-to-Cue Task; WCST = Wisconsin Card Sorting Test; FIST = Flexible Item Selection Task

We tested our hypotheses using two approaches, both for each sample separately and in the combined sample (i.e., Samples 1 and Sample 2 combined). First, we inspected the correlations between the manifest scores from the executive function tasks and the emotional granularity indices. We corrected for multiple testing by applying Benjamini and Hochberg’s (1995) procedure for controlling for the false discovery rate (FDR) across the six significance tests of the correlations for negative and positive emotional granularity separately. Second, we tested the associations between executive functions and emotional granularity using a latent variable approach. To identify a suitable measurement model of executive functions, we conducted confirmatory factor analyses with the manifest task scores as indicator variables in *lavaan* (version 0.6–17; Rosseel, 2012), using full information maximum likelihood (FIML) estimation with robust (Huber-White) standard errors (MLR). An expected three-factor model, in which the executive function task scores loaded on specific executive function domain factors, yielded non-positive definite covariance matrices for the latent variables in each sample and in the combined sample. In contrast, an alternative single-factor model, in which all six executive function task scores loaded on a common executive function factor, showed a good model fit, χ^2^(*df* = 9, *N* = 153) = 12.50, *p* = .187, CFI = .987, RMSEA = .044, SRMR = .050 in Sample 1, χ^2^(*df* = 9, *N* = 218) = 10.86, *p* = .285, CFI = .991, RMSEA = .038, SRMR = .033 in Sample 2, and χ^2^(*df* = 9, *N* = 371) = 15.50, *p* = .078, CFI = .983, RMSEA = .052, SRMR = .031 in the combined sample. We therefore selected this model as the final measurement model for executive function. For the single-factor model, partially weak measurement invariance (i.e., equal factor loadings for all but one indicator) was found to hold across the two samples (for details on measurement model selection and the tests of measurement invariance, see Chapter 2 and Tables S2 and S3 in the Supplementary Material). In the next steps, we incorporated the two emotional granularity indices into the model (see Fig. 1) and estimated their relations with the latent executive function factor.^1^ Because we hypothesized positive correlations between executive functions and both negative and positive emotional granularity, we interpret one-tailed *p*-values of the respective significance tests of both manifest and latent correlations between executive functions and emotional granularity (Hales, 2024).

### Sample Size Considerations

The present study was part of a larger research project with multiple research questions. The project aimed for a total sample size of 150 participants with and 150 without chronic pain. For the preregistration of the present research, we conducted a sensitivity power analysis using G*Power (Faul et al., 2009) and found that a sample size of *N* = 150 allowed us to detect a correlation of .20 for a one-tailed test with a power of .80 (α = .05). By combining the two samples into a larger total sample (*N* = 371), the power to detect a correlation of .20 for a one-tailed test (α = .05) increased to .99. Additionally, we conducted Monte Carlo simulation studies to determine the power to detect small to moderate latent correlations between executive functions and emotional granularity using the shiny app *pwrSEM* with 1,000 replications for each simulation (Wang & Rhemtulla, 2021; https://yilinandrewang.shinyapps.io/pwrSEM/). For the preregistration, we assumed a three-factor measurement model of executive functions with each executive function domain measured by two task score indicators with standardized loadings of 0.65, based on a sample of *N* = 150. The power estimates for detecting a correlation between an executive function domain factor and a manifest emotional granularity index were .58, .71, and .84 for correlation sizes of .20, .25, and .30, respectively, for one-tailed tests (α = .05). Revisiting the simulation studies post hoc for the combined sample (*N* = 371) and specifying a single-factor measurement model of executive functions with standardized loadings of 0.65 for all executive function task indicators, we found a power estimate of .98 for detecting a correlation of .20 between the overall executive function factor and a manifest emotional granularity index. Overall, we concluded that our study was sufficiently powered to detect small to medium-sized correlations between executive functions and emotional granularity within each sample and in the combined sample.

## Results

### Descriptive Statistics

Descriptive statistics for the variables under study in Samples 1 and 2, as well as in the combined sample, are presented in Table 1, along with the results of *t-*tests for investigating differences between Samples 1 and 2. Whereas we found significant differences between Samples 1 and 2 in positive emotional granularity, mean negative emotions, and mean positive emotions, with Sample 1 (i.e., individuals without chronic pain) showing higher positive emotional granularity, lower mean intensity levels of negative emotions, and higher mean intensity levels of positive emotions than Sample 2 (i.e., individuals with chronic pain), there were no significant differences in the other study variables.

**Table 1:** Descriptive statistics for study variables in Samples 1 and 2 and the combined sample, and statistical tests for mean differences between Samples 1 and 2

Variable	Sample 1			Sample 2			Combined sample			Mean differences between Samples 1 and 2		
	*M*	*SD*	*n*	*M*	*SD*	*n*	*M*	*SD*	*n*	|*t*|	*p* (two-tailed)	|*d*|
VCST	83.03	10.30	148	82.67	10.09	215	82.82	10.16	363	0.33	.741	0.04
SNT	85.55	7.63	150	85.54	6.76	214	85.54	7.12	364	0.01	.995	0.00
AST	79.53	14.44	143	76.48	15.79	206	77.73	15.30	349	1.86	.063	0.20
SACT	85.01	15.16	147	82.93	14.67	207	83.79	14.89	354	1.29	.197	0.14
WCST	81.44	7.15	145	79.92	9.56	211	80.54	8.69	356	1.72	.087	0.18
FIST	60.86	21.63	151	62.34	20.44	218	61.73	20.92	369	0.66	.509	0.07
Neg. EG	–1.24	0.39	153	–1.31	0.35	218	–1.28	0.37	371	1.56	.120	0.17
Pos. EG	–1.26	0.29	153	–1.35	0.27	218	–1.31	0.28	371	2.86	.004	0.30
*M* NE	9.87	10.87	153	14.22	11.29	218	12.43	11.31	371	3.73	< .001	0.39
*M* PE	59.17	13.49	153	51.61	14.09	218	54.73	14.32	371	5.22	< .001	0.55

VCST = Visual-Verbal Complex Span Task; SNT = Spatial *n*-Back Task; AST = Antisaccade Task; SACT = Sustained Attention-to-Cue Task; WCST = Wisconsin Card Sorting Test; FIST = Flexible Item Selection Task. Neg. EG = negative emotional granularity index; Pos. EG = positive emotional granularity index; *M* NE = mean negative emotions; *M* PE = mean positive emotions

### Manifest Correlations Between Executive Function Tasks and Emotional Granularity Indices

Correlations between executive function task scores and the emotional granularity indices in the two samples and in the combined sample are presented in Table 2 (for correlations between all study variables, see Table S1 in Supplementary Material). For the negative emotional granularity index, correlations with the executive function task scores ranged from –.04 to .10 in Sample 1, from –.14 to .11 in Sample 2, and from –.10 to .08 in the combined sample, with all one-tailed *p*-values > .05. For the positive emotional granularity index, correlations with the executive function task scores ranged from –‍.17 to .03 in Sample 1, from –.01 to .12 in Sample 2, and from –.07 to .06 in the combined sample, with all non-significant after FDR correction. Taken together, correlations at the manifest level in each sample and in the combined sample did not support our hypotheses that executive function domains would be positively associated with negative and positive emotional granularity.

**Table 2:** Correlations between executive function task scores and negative and positive emotional granularity indices in Samples 1 and 2 and the combined sample

Variable	Sample 1		Sample 2		Combined sample	
	*r*	*p*	*r*	*p*	*r*	*p*
Negative emotional granularity						
VCST	–.03	.654	–.05	.763	–.04	.779
SNT	–.00	.511	–.06	.807	–.03	.733
AST	.04	.323	.01	.427	.03	.275
SACT	.10	.116	.03	.343	.06	.113
WCST	.02	.397	.11	.058	.08	.056
FIST	–.04	.691	–.14	.983	–.10	.972
Positive emotional granularity						
VCST	–.15	.969	.06	.203	–.03	.715
SNT	–.12	.923	.02	.361	–.04	.773
AST	–.17	.976	.12	.037^a^	.02	.326
SACT	–.05	.741	.01	.460	–.01	.570
WCST	.03	.340	.05	.241	.06	.143
FIST	–.13	.949	–.01	.580	–.07	.911

Given that our hypotheses on the relation between executive functions and emotional granularity indices were directional (ρ > 0), the reported *p*-values are one-tailed *p*-values. Correlations between executive function tasks are reported in Table S1 in the Supplementary Material. VCST = Visual-Verbal Complex Span Task; SNT = Spatial *n*-Back Task; AST = Antisaccade Task; SACT = Sustained Attention-to-Cue Task; WCST Wisconsin Card Sorting Test; FIST = Flexible Item Selection Task

^a^ *p*-value not under critical threshold after false discovery correction

#### Latent Correlations Between Executive Function Tasks and Emotional Granularity Indices

Figure 1 depicts the results of the latent variable mode in Sample 1 (Panel a), Sample 2 (Panel b), and the combined sample (Panel c). The negative emotional granularity index showed no significantly positive association with the common executive function factor in Sample 1, *r* = .00, *z* = 0.01, one-tailed *p* = .495, 95% CI [–.14, .14], Sample 2, *r* = –.06, *z* = –0.77, one-tailed *p* = .779, 95% CI [–.21, .09], or the combined sample, *r* = –.03, *z* = –0.53, one-tailed *p* = .702, 95% CI [–.14, .08]. Moreover, the positive emotional granularity index showed no significantly positive association with the common executive function factor in Sample 1, *r* = –.16, *z* = –1.59, one-tailed *p* = .945, 95% CI [–.35, .02], Sample 2, *r* = .06, *z* = 0.89, one-tailed *p* = .186, 95% CI [–.07, .19], or the combined sample, *r* = –.03, *z* = –0.54, one-tailed *p* = .706, 95% CI [–.15, .08]. Taken together, in line with the correlations at the manifest level, the correlations at the latent level did not support our hypotheses that executive function domains would be positively associated with negative and positive emotional granularity.

### Bayes Factors

Given that non-significant findings in null hypothesis significance testing do not provide evidence for the absence of a meaningful effect, we cannot conclude from the results of our significance tests that there were no substantial positive associations between executive functions and (negative/positive) emotional granularity. To better understand whether our findings suggest a lack of meaningful positive associations between executive functions and emotional granularity, we additionally calculated Bayes factors (BFs_12_; Dienes, 2014; Hoijtink et al., 2019). Specifically, by means of BFs_12_, we tested the extent to which our data provided sufficient evidence for hypothesis H_1_ that the correlation between executive functions and emotional granularity is below .20 compared to hypothesis H_2_ that the correlation is larger than or equal to .20. We chose the threshold of ρ = .20 as we considered correlations under this threshold to not represent practically meaningful positive associations between the variables and because it corresponded to the minimum effect size of interest in our power analyses. A BF_12_ value > 1 indicates more evidence for H_1_ than for H_2_. Following Jeffreys (1961), we interpreted BF_12_ values > 3.2 as moderate evidence and values > 10 as strong evidence for H_1_. For the manifest correlations, we calculated BFs_12_ using the *BFpack* package (version 1.4.0; Mulder et al., 2021). For the latent correlations, we used the *bain* package (version 0.2.10) based on the tutorial by Van Lissa et al. (2021). In both analyses, we used default uninformative priors. These analyses were not preregistered.

Table 3 presents the median BF_12_ values (and their range) computed across the six manifest correlations and the BF_12_ values for the latent correlations for negative and positive emotional granularity separately, in each sample and the combined sample. For simplification, we report extremely high values (i.e., greater than 100) as “ > 100”. All BF_12_ values indicated at least moderate, but mostly strong evidence in favor of H_1_ in each sample and the combined sample and for both negative and positive emotional granularity. Therefore, our data provided substantial evidence for a lack of meaningful positive associations between executive functions and emotional granularity.

**Table 3:** Bayes factors for manifest and latent correlations between executive functions and negative and positive emotional granularity indices in Samples 1 and 2 and the combined sample

EG valence	Sample 1		Sample 2		Combined sample	
	Manifest correlations: Median BF_12_ (range)	Latent correlation with EF factor: BF_12_	Manifest correlations: Median BF_12_ (range)	Latent correlation with EF factor: BF_12_	Manifest correlations: Median BF_12_ (range)	Latent correlation with EF factor: BF_12_
Negative EG	67.78 (5.64; > 100)	> 100	> 100 (6.72; > 100)	> 100	> 100 (48.09; > 100)	> 100
Positive EG	> 100 (28.92; > 100)	> 100	92.41 (4.35; > 100)	59.26	> 100 (> 100; > 100)	> 100

EG = emotional granularity; EF = executive function; BF_12_ = Bayes factor for evidence in favor of hypothesis H_1_ (correlation is below .20) compared to evidence in favor of hypothesis H_2_ (correlation is larger than or equal to .20)

### Supplementary Analyses

We conducted supplementary analyses to test the robustness of our findings. First, we explored whether the associations between executive functions and emotional granularity were affected by controlling for mean levels of negative and positive emotions. These analyses accounted for the possibility that granularity values are bounded by the intensity with which individuals experience emotions (e.g., individuals may have low negative emotional granularity values just because they experience negative emotions at very high levels; Dejonckheere et al., 2019). At the manifest level (i.e., using the manifest executive function scores as predictors), we regressed the negative and positive emotional granularity indices on the executive function task scores and either the mean negative or positive emotion scores in separate multiple regression models for each executive function task score. For the models in which negative emotional granularity was the criterion, the mean negative emotion score was included as a control, and for the models with positive emotional granularity, the mean positive emotion score was used as the control. At the latent level, the negative emotional granularity index was regressed on the common executive function factor and the mean negative emotion score, while the positive emotional granularity index was regressed on the common executive function factor and the mean positive emotion score, in a single latent variable model. These analyses were not preregistered. At neither the manifest nor the latent level did we find significant positive associations between executive functions and emotional granularity when controlling for mean levels of emotions (see Tables S4 for analyses at the manifest level and S5 for analyses at the latent level in the Supplementary Material for partial regression coefficients of executive functions in predicting negative or positive emotional granularity). Hence, our null findings remained unchanged after statistically controlling for mean levels of emotions.

Second, we explored whether the associations between the executive function task scores and the emotional granularity indices were moderated by age, given the wide age range in our samples (i.e., 14–83 years). To do this, we regressed the negative and positive emotional granularity indices on the executive function task scores (separately for each of the six task scores), along with a linear and quadratic term for age and their interactions with the executive function task scores. We included a quadratic term for age because age has been shown to exhibit quadratic associations with executive functions (Ferguson et al., 2021) and emotional granularity (Nook et al., 2018). Since we had no specific hypotheses regarding the direction of linear or quadratic moderating effects of age, we used two-tailed *p*-values for these analyses. These analyses were not preregistered. The results are presented in Tables S6–S11 of the Supplementary Material. Apart from a significant quadratic moderating effect of age on the association between the SNT score and positive emotional granularity in Sample 1, no significant moderating effects of age were found in any of the samples (i.e., in 35 out of a total of 36 analyses). Therefore, we deemed our findings largely robust to potential moderator effects of age.

Third, as preregistered, we explored whether there were unique positive associations between executive function task scores and the emotional granularity indices when controlling for all other executive function task scores (i.e., by regressing the negative and positive emotional granularity indices on all executive function task scores simultaneously). None of the partial regression coefficients for the executive function task scores were significantly greater than zero in any of the samples (see Table S12 in Supplementary Material), indicating that there were no unique positive associations between executive function tasks and emotional granularity.

Fourth, we analyzed associations between percentages of specific types of errors available for the SNT (omissions and false alarms) and the WCST (perseveration and drift errors) and emotional granularity indices. These analyses were not preregistered. Note that higher error scores correspond to lower executive function performance. In line with results from the main analyses using overall percentage correct scores, we did not find significant negative correlations between these specific error types and emotional granularity (see Table S13 in the Supplementary Material for correlation coefficients).

Fifth, our item sets for negative and positive emotions consisted of items that could be grouped into different emotion categories (e.g., *sad*, *downhearted*, and *unhappy* for the category “sadness”; *ashamed*, *humiliated*, and *disgraced* for “shame”). As preregistered, we additionally computed indices of emotional granularity to assess granularity between broader emotion categories (i.e., between-category emotional granularity indices) and between emotions that fell under the same category (i.e., within-category emotional granularity indices; Erbas et al., 2019). At neither the manifest nor latent level did we find any significant positive associations between executive function task scores and indices of between-category or within-category (negative/positive) emotional granularity (see Chapter 7 of Supplementary Material together with Tables S14 and S15 for more details), indicating that there were also no meaningful positive associations between executive functions and emotional granularity for different levels of specificity at which emotions are differentiated.

## Discussion

The present preregistered study investigated the associations between different executive function domains (i.e., working memory, inhibition, and shifting) and both negative and positive emotional granularity. It combined an online assessment of executive functions, using six cognitive tasks designed to measure the three executive function domains, with a 14-day ambulatory assessment of participants’ emotional experiences and included two samples (i.e., individuals with and without chronic pain). Contrary to our hypotheses, we found no significant positive associations between executive functions and negative or positive emotional granularity at the manifest level (i.e., using the six manifest executive function task scores) or at the latent level (i.e., using a common executive function factor measured by the executive function task score indicators), and this was the case both for each sample and in analyses combining both samples. Importantly, several supplementary analyses (e.g., calculation of Bayes factors, controlling for mean emotion scores, using alternative emotional granularity indices that take into account the hierarchical structure of our emotion item set) consistently supported the robustness of our findings.

Our findings contrast with previous theoretical considerations that executive functions should facilitate a granular categorization of individuals’ emotional experiences by coordinating the integration of affect-related information from the environment and the body with appropriate emotion concepts (Lee et al., 2017; Lindquist & Barrett, 2008). Furthermore, they are inconsistent with an unpublished study (Barrett et al., as cited in Lee et al., 2017) that observed a positive relationship between working memory and negative emotional granularity. Compared to this unpublished study, our study assessed executive functions and emotional granularity in a more nuanced way. On the one hand, it included a broad array of different executive function tasks to directly assess all three domains of executive functions (Miyake et al., 2000). On the other hand, it investigated the link between executive functions and emotional granularity for both negative and positive emotional granularity separately. Therefore, we consider our study to be a valuable contribution to the sparse literature on a potential link between executive functions and emotional granularity.

Presumably, alternative, less common operationalizations of emotional granularity may capture individual differences in emotional granularity that are more closely associated with individual differences in executive functions. For example, laboratory-based tasks involving repeated emotion ratings to novel emotional stimuli (e.g., Erbas et al., 2019) require participants to match unfamiliar affect-related input with emotion concepts accrued from past experiences. This may be more cognitively effortful than providing emotion ratings in daily life, where participants are exposed to more familiar affect-related input. Moreover, granularity assessed through the coding of participants’ open-ended descriptions of their emotional experiences may be more closely associated with executive functions than assessed through the use of closed-ended lists of emotion items provided by the researcher (Ottenstein & Lischetzke, 2020). Finding one’s own words to describe one’s emotional experiences in a precise manner necessitates an active and deliberate search for the most appropriate emotional concepts, which is likely to require more in-depth processing than the mere endorsement of pre-defined emotion items (Hoemann et al., 2024). Hence, the link between executive functions and emotional granularity may depend on the cognitive demands inherent to the various operationalizations of emotional granularity. Future research may explore this assumption.

One limitation of our study is that our executive function tasks may not have tapped into those aspects of executive functions that are relevant to emotional granularity. “Cold” executive functions, which involve purely cognitive information processing in neutral, low-stake situations, have been differentiated from “hot” executive functions, which involve affect-related information processing in motivationally or emotionally salient high-stake situations that provide cues for threats or rewards (Salehinejad et al., 2021; Zelazo & Carlson, 2012). As the categorization of emotions requires individuals to process affect-related input in emotional situations, it may primarily rely on hot executive functions. Furthermore, emotional granularity has been linked to greater cortical thickness of the lateral orbitofrontal cortex, a brain region implicated in the controlled selection of emotion concepts (Lukic et al., 2023). This same region has also been associated with hot executive functions, whereas cold executive functions tend to involve more dorsolateral and lateral regions of the prefrontal cortex (Salehinejad et al., 2021; Zelazo & Carlson, 2012). Therefore, both conceptual considerations and neuroanatomical insights suggest that hot executive functions may play a stronger role than cold executive functions in supporting emotional granularity. However, our executive function tasks likely tapped primarily cold aspects of executive functions, as they used neutral stimuli (e.g., animals or flowers) devoid of emotional context. Future research could address this limitation by including emotionally salient stimuli (e.g., happy vs. sad faces) in the executive function tasks or administering executive function tasks in more high-stake situations (e.g., by implementing rewards).

Another potential limitation is the web-based administration of executive function tasks, which may have affected internal validity due to external influences beyond the researchers’ control (e.g., distraction). However, research has shown that web-based and laboratory-based cognitive assessments yield comparable results (Germine et al., 2012; Uittenhove et al., 2023), suggesting our findings would likely be similar in a lab setting.

## Conclusion

Our study does not provide evidence for positive associations between executive functions and negative or positive emotional granularity, challenging theoretical perspectives about the cognitive mechanisms of emotional granularity. Future research using different operationalizations of emotional granularity and/or assessing hot executive functions could explore whether executive functions and emotional granularity are associated through mechanisms not captured in the present study.

## Supplementary Information

Below is the link to the electronic supplementary material.

Supplementary file1 (PDF 473 KB)

## Footnotes

1 We originally preregistered separate models for the correlations of the EF factor with the negative and positive EG indices. However, for simplicity, we report a full model including both correlations simultaneously. This did not impact the results.

## Additional Information

### Data availability

Data relevant to the analysis and analysis scripts for this research can be accessed via https://osf.io/usvcz/.
