# Supplementary material for: Executive Functions and Emotional Granularity: No Evidence for Positive Associations
Source: Affect Sci. 2025 May 23;6(3):464–76. doi: 10.1007/s42761-025-00307-y (PMC12579614; doi:10.1007/s42761-025-00307-y)
Supplement: Supplementary file 1 — Supplementary file1 (PDF 473 KB) [file 42761_2025_307_MOESM1_ESM.pdf]

Supplementary Material

Executive functions and emotional granularity: No evidence for positive associations

Table of contents

1. Correlations between study variables 3

2. Measurement model selection process for executive functions 4

3. Supplementary analyses: Controlling for mean emotions 6

4. Supplementary analyses: Moderating effects of age 7

5. Exploratory results for regressing negative and positive emotional granularity indices on all executive function task scores simultaneously 13

6. Correlations between specific error scores of executive function tasks and emotional granularity indices 14

7. Relations of executive functions with between-category and within-category indices of negative and positive emotional granularity 15

8. References 19

1. Correlations between study variables

Table S1 presents correlations between all study variables in both and the combined samples.

Table S1

Correlations between study variables in Samples 1 and 2 and the combined sample

Variable	1	2	3	4	5	6	7	8	9
				Sample 1					
1. VCST	–								
2. SNT	.35***	–							
3. AST	.22**	.41***	–						
4. SACT	.24**	.42***	.29***	–					
5. WCST	.12	.40***	.22*	.29***	–				
6. FIST	.50***	.55***	.36***	.29***	.33***	–			
7. Mean NE	.03	–.21**	–.02	–.17*	–.24**	.02	–		
8. Mean PE	–.02	.06	–.15	.05	.12	.08	–.42***	–	
9. Negative EG	–.03	.00	.04	.10	.02	–.04	–.47***	.29***	–
10. Positive EG	–.15	–.12	–.17**	–.05	.03	–.13	–.04	.12	.31***
				Sample 2					
1. VCST	–								
2. SNT	.40***	–							
3. AST	.41***	.34***	–						
4. SACT	.20**	.30***	.38***	–					
5. WCST	.34***	.37***	.28**	.26***	–				
6. FIST	.55***	.44***	.51***	.30***	.40***	–			
7. Mean NE	.05	.01	–.05	–.09	–.11	.02	–		
8. Mean PE	–.06	–.01	–.04	.09	–.08	–.12	–.37***	–	
9. Negative EG	–.05	–.06	.01	.03	.11	–.14*	–.47***	.13	–
10. Positive EG	.06	.02	.12	.01	.05	–.01	–.06	.00	.35***
				Combined sample					
1. VCST	–								
2. SNT	.38***	–							
3. AST	.33***	.37***	–						
4. SACT	.22***	.35***	.35***	–					
5. WCST	.27***	.38***	.26***	.26***	–				
6. FIST	.53***	.49***	.45***	.29***	.36***	–			
7. Mean NE	.04	–.09	–.06	–.13*	–.17**	.03	–		
8. Mean PE	–.04	.02	–.05	.09	.01	–.04	–.42***	–	
9. Negative EG	–.04	–.03	.03	.06	.08	–.10	–.47***	.21	–
10. Positive EG	–.03	–.04	.02	–.01	.06	–.07	–.08	.08	.34***

Note. For reasons of consistency within the table, asterisks refer to two-tailed *p*-values below .05 for all correlations. For correlations that were part of our hypotheses (i.e., correlations between executive function task scores and emotional granularity), we interpret one-tailed *p*-values (see Table 2 in the manuscript). VCST = Visual-Verbal Complex Span Task; SNT = Spatial *n*-Back Task; AST = Antisaccade Task; SACT = Sustained Attention-to-Cue Task; WCST = Wisconsin Card Sorting Test; FIST = Flexible Item Selection Task; Mean NE = mean negative emotions; Mean PE = mean positive emotions; EG = emotional granularity.

* $p < .05$ (two-tailed). ** $p < .01$ (two-tailed). *** $p < .001$ (two-tailed).

2. Measurement model selection process for executive functions

To find an appropriate measurement model for executive functions in both samples, we conducted confirmatory factor analyses with the manifest task scores as indicator variables, using *lavaan* (version 0.6-17; Rosseel, 2012). We used a robust maximum likelihood estimator ("MLR") to account for non-normally distributed manifest indicator variables. Missing data were handled by specifying full information maximum likelihood (FIML) estimation.

Following the work of Miyake et al. (2000), we first specified a three-factor model, such that each executive function domain represented a factor measured by the respective executive function task scores. However, this model resulted in a non-positive definite matrix covariance matrix of the latent variables in both samples. The same outcome occurred in a nested factor model, in which all task scores loaded on a common executive function factor, while the working memory and shifting task scores additionally loaded on a specific working memory and shifting factor, respectively, both orthogonal to each other and the common executive function factor (Miyake & Friedman, 2012).

We then specified a simpler single-factor level in which all six task scores loaded only on a common executive function factor. This model yielded a good model fit, $\chi^2(df = 9, N = 153) = 12.50$, $p = .187$, CFI = .97, RMSEA = .05, SRMR = .05 for Sample 1 and $\chi^2(df = 9, N = 218) = 10.86$, $p = .285$, CFI = .990, RMSEA = .031, SRMR = .033 for Sample 2. We further examined whether accounting for common variance across tasks that were administered in the same day block improved model fit by adding an additional orthogonal indicator-specific factor on which only the task scores of the second day block (i.e., SNT, SACT, and FIST) loaded. However, this did not significantly improve model fit for Sample 1, $\chi^2(df = 1, N = 153) = 0.07$, $p = .793$, and resulted in an uninterpretable model with negative latent variances for Sample 2. We selected the simple one-factor model as the final measurement model of executive function and as the basis of our analytic model (which included the correlations with the EG indices) for both samples.

We tested whether the selected single-factor model was invariant across the two samples. Table S2 presents the absolute fit indices of the different models that we compared, and Table S3 shows the results of the model comparisons. Equating the factor loadings (weak measurement invariance) led to substantial changes in some model fit indices compared to a configural measurement invariance model (e.g., ΔCFI < –.005; ΔRMSEA > .01; Chen, 2007), indicating that full weak measurement invariance was not met. Based on modification indices, we allowed the loadings of the SNT to vary while keeping all other loadings equal across samples (partially weak measurement invariance). Compared to the configural measurement invariance model, the partially weak measurement invariance model did not lead to a substantially worse model fit. In the next step, we equated the indicator intercepts, except for those of the

SNT, across the samples (partially strong measurement invariance). However, this model had a worse fit than the partially weak measurement invariance model (e.g., ΔCFI < –.005; ΔRMSEA > .01). Taken together, partially weak measurement invariance held across the two samples, suggesting that the relations between the latent executive function factor and its task score indicators are largely equivalent across samples.

Table S2

Absolute fit indices of measurement invariance models

Model	*df*	χ^2	*p* value (χ^2)	CFI	RMSEA	SRMR	MNCI	AIC	BIC
configural	18	23.30	.179	.989	.041	.040	.986	16391.62	16532.39
weak	23	30.52	.135	.978	.052	.069	.976	16394.72	16516.12
**partially weak**	**22**	**23.92**	**.351**	**.994**	**.028**	**.055**	**.986**	**16388.04**	**16513.36**
partially strong	26	31.66	.205	.986	.039	.059	.979	16388.94	16498.59

Note. In the partially weak model, the loadings of the SNT were allowed to vary across samples. In the partially strong model, the loadings and the intercepts of the SNT were allowed to vary across samples.

Table S3

Relative fit indices of measurement invariance models

Model	Δ*df*	$\Delta\chi^2$	*p* value ($\Delta\chi^2$)	ΔCFI	ΔRMSEA	ΔSRMR	ΔMNCI
weak – configural	5	7.02	.219	–.011	.011	.029	–.010
partially weak – configural	4	2.15	.708	.005	–.13	.015	0
partially strong – partially strong	4	8.69	.069	–.008	.011	.004	–.007

3. Supplementary analyses: Controlling for mean emotions

Table S4

Partial regression coefficients of manifest executive function task scores for predicting negative and positive emotional granularity when controlling for mean levels of negative or positive emotions

Task score	Sample 1			Sample 2			Combined sample		
	B	*p*	β	*B*	*p*	β	*B*	*p*	β
Negative emotional granularity									
VCST	–0.001	.604	–.019	–0.001	.649	–.023	–0.001	.679	–.021
SNT	–0.005	.925	–.107	–0.003	.812	–.054	–0.004	.944	–.074
AST	0.001	.343	.029	0.000	.568	–.011	0.000	.473	.003
SACT	0.000	.444	.010	0.000	.576	–.012	0.000	.497	.000
WCST tot	–0.005	.889	–.093	0.002	.183	.055	0.000	.454	.005
FIST	–0.001	.671	–.032	–0.002	.987	–.134	–0.002	.972	–.088
Positive emotional granularity									
VCST	–0.004	.968	–.153	0.002	.202	.057	–0.001	.692	–.026
SNT	–0.005	.934	–.123	0.001	.362	.024	–0.002	.782	–.041
AST	–0.003	.959	–.146	0.002	.038[a]	.124	0.001	.292	–.029
SACT	–0.001	.765	–.060	0.000	.461	.007	0.000	.628	–.017
WCST tot	0.001	.396	.022	0.001	.246	.048	0.002	.144	.056
FIST	–0.002	.961	–.144	0.000	.583	–.014	–0.001	.900	–.067

Note. Each executive function score was used separately as a predictor, along with either mean negative emotions (for predicting negative emotional granularity) or mean positive emotions (for predicting positive emotional granularity). Given that our hypotheses on the relationships between executive functions and emotional granularity indices were directional, the reported *p*-values are one-tailed *p*-values. VCST = Visual-Cerbal Complex Span Task; SNT = Spatial *n*-Back Task; AST = Antisaccade Task; SACT = Sustained Attention-to-Cue Task; WCST = Wisconsin Card Sorting Test; FIST = Flexible Item Selection Task.

[a] *p*-value not under critical threshold after false discovery correction.

Table S5

Partial regression coefficients of latent common executive function factor for predicting negative and positive emotional granularity when controlling for mean levels of negative or positive emotions

Criterion	Sample 1			Sample 2			Combined sample		
	B	*p*	β	*B*	*p*	β	*B*	*p*	β
Negative EG	–0.006	.848	–.098	–0.004	.826	–.069	–0.004	.903	–.072
Positive EG	–0.008	.959	–.163	0.003	.174	.063	–0.001	.684	–.028

Note. For negative emotional granularity, mean negative emotions was used as a control variable, for positive emotional granularity, mean positive emotions was used as a control variable. Given that our hypotheses on the relationships between executive functions and emotional granularity indices were directional, the reported *p*-values are one-tailed *p*-values. EG = emotional granularity.

4. Supplementary analyses: Moderating effects of age

Table S6

Partial regression coefficients for testing moderating effects of age on the association between VCST and emotional granularity

Variable	Negative emotional granularity			Positive emotional granularity		
	B	*p* (two-tailed)	β	*B*	*p* (two-tailed)	β
			Sample 1			
Intercept	–1.216	–	–	–1.296	–	–
Age	0.047	.061	.187	–0.010	.576	–.054
Age sq	–0.014	.324	–.085	0.023	.027	.188
VCST	0.002	.584	.058	–0.005	.081	–.183
VCST x Age	0.000	.965	.004	0.004	.087	.199
VCST x Age sq	–0.001	.608	–.038	–0.000	.894	–.010
			Sample 2			
Intercept	–1.322	–	–	–1.392	–	–
Age	0.036	.037	.162	–0.010	.462	–.057
Age sq	0.007	.481	.051	0.023	.003	.215
VCST	0.003	.480	.079	–0.001	.752	–.035
VCST x Age	0.002	.281	.081	0.001	.607	.038
VCST x Age sq	–0.001	.356	–.071	0.001	.247	.088
			Combined sample			
Intercept	–1.277	–	–	–1.350	–	–
Age	0.040	.005	.169	–0.007	.519	–.038
Age sq	–0.001	.892	–.007	0.021	.001	.185
VCST	0.002	.421	.060	–0.003	.159	–.105
VCST x Age	0.001	.325	.061	0.002	.167	.085
VCST x Age sq	–0.001	.298	–.053	0.001	.230	.061

Note. Age in years was divided by 10 (such that one unit corresponds to one decade) in order to increase regression coefficients. Age sq = age squared; VCST = Visual-Verbal Complex Span Task.

Table S7

Partial regression coefficients for testing moderating effects of age on the association between SNT and emotional granularity

Variable	Negative emotional granularity			Positive emotional granularity		
	B	*p* (two-tailed)	β	*B*	*p* (two-tailed)	β
			Sample 1			
Intercept	–1.221	–	–	–1.286	–	–
Age	0.053	.026	.210	–0.003	.843	–.018
Age sq	–0.014	.326	–.084	0.007	.479	.060
SNT	0.003	.605	.061	–0.011	.013	–.289
SNT x Age	–0.001	.634	–.043	–0.005	.039	–.185
SNT x Age sq	–0.000	.838	–.014	0.002	.050	.137
			Sample 2			
Intercept	–1.328	–	–	–1.395	–	–
Age	0.034	.040	.154	–0.014	.301	–.078
Age sq	0.004	.668	.034	0.022	.007	.198
SNT	0.001	.913	.015	0.003	.466	.083
SNT x Age	–0.002	.338	–.071	0.003	.183	.100
SNT x Age sq	–0.000	.932	–.008	–0.001	.549	–.043
			Combined sample			
Intercept	–1.282	–	–	–1.346	–	–
Age	0.042	.002	.179	–0.009	.386	–.051
Age sq	–0.004	.647	–.025	0.015	.018	.130
SNT	0.001	.726	.028	–0.005	.149	–.116
SNT x Age	–0.002	.277	–.061	–0.001	.552	–.034
SNT x Age sq	–0.000	.885	–.007	0.000	.154	.070

Note. Age in years was divided by 10 (such that one unit corresponds to one decade) in order to increase regression coefficients. Age sq = age squared; SNT = Spatial *n*-Back Task.

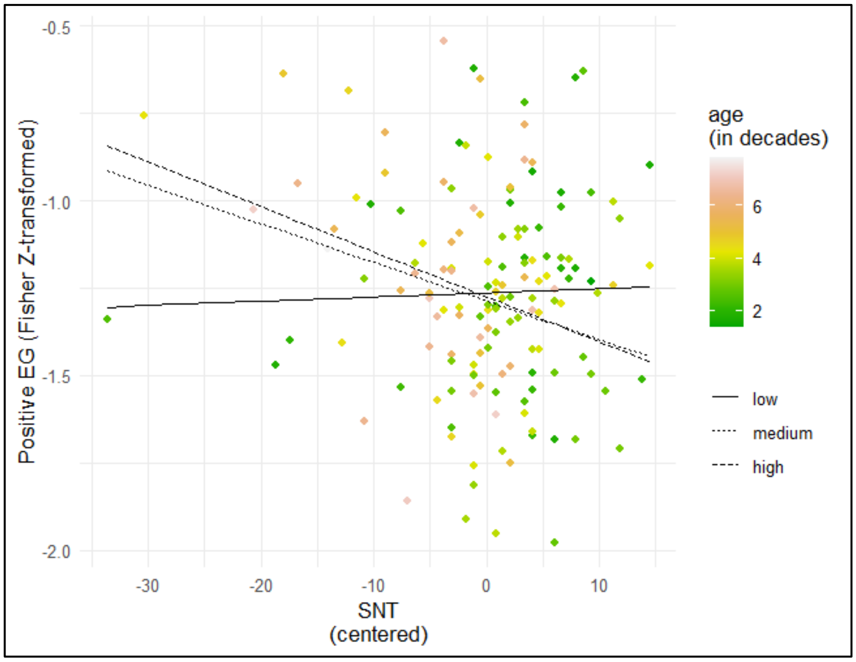

Fig. S1 Simple slopes for association between SNT and positive emotional granularity moderated by age for Sample 1 (individuals without chronic pain)

Table S8

Partial regression coefficients for testing moderating effects of age on the association between AST and emotional granularity

Variable	Negative emotional granularity			Positive emotional granularity		
	B	*p* (two-tailed)	β	*B*	*p* (two-tailed)	β
			Sample 1			
Intercept	–1.245	–	–	–1.307	–	–
Age	0.066	.006	.262	0.001	.945	.006
Age sq	0.001	.958	.005	0.025	.031	.202
AST	0.004	.242	.137	–0.002	.466	–.086
AST x Age	–0.001	.556	–.060	0.002	.205	.125
AST x Age sq	0.000	.848	.013	–0.000	.601	–.035
			Sample 2			
Intercept	–1.330	–	–	–1.402	–	–
Age	0.045	.014	.202	–0.006	.684	–.033
Age sq	0.003	.790	.020	0.022	.009	.199
AST	0.003	.284	.121	0.002	.360	.103
AST x Age	–0.002	.098	–.138	–0.000	.731	–.029
AST x Age sq	0.000	.813	.019	0.000	.520	.053
			Combined sample			
Intercept	–1.292	–	–	–1.363	–	–
Age	0.055	.000	.231	–0.003	.764	–.183
Age sq	0.001	.900	.008	0.023	.001	.200
AST	0.003	.077	.140	0.001	.373	.071
AST x Age	–0.002	.112	–.100	0.001	.419	.051
AST x Age sq	0.000	.799	.014	–0.000	.842	–.011

Note. Age in years was divided by 10 (such that one unit corresponds to one decade) in order to increase regression coefficients. Age sq = age squared; AST = Antisaccade Task.

Table S9

Partial regression coefficients for testing moderating effects of age on the association between SACT and emotional granularity

Variable	Negative emotional granularity			Positive emotional granularity		
	B	*p* (two-tailed)	β	*B*	*p* (two-tailed)	β
			Sample 1			
Intercept	–1.237	–	–	–1.299	–	–
Age	0.043	.061	.168	0.003	.877	.014
Age sq	–0.005	.723	–.028	0.015	.139	.118
SACT	–0.001	.878	–.020	0.000	.877	.022
SACT x Age	–0.003	.044	–.177	0.000	.702	.034
SACT x Age sq	0.001	.141	.112	–0.000	.599	–.041
			Sample 2			
Intercept	–1.335	–	–	–1.407	–	–
Age	0.043	.015	.187	–0.016	.235	–.091
Age sq	0.014	.218	.093	0.028	.001	.246
SACT	0.000	.863	.017	–0.002	.301	–.102
SACT x Age	–0.001	.692	–.034	–0.002	.089	–.146
SACT x Age sq	0.001	.386	.057	0.001	.024	.148
			Combined sample			
Intercept	–1.292	–	–	–1.357	–	–
Age	0.045	.001	.186	–0.009	.387	–.050
Age sq	0.004	.599	.028	0.021	.001	.173
SACT	0.001	.751	.025	–0.001	.627	–.038
SACT x Age	–0.002	.086	–.104	–0.001	.456	–.046
SACT x Age sq	0.001	.136	.071	0.000	.285	.052

Note. Age in years was divided by 10 (such that one unit corresponds to one decade) in order to increase regression coefficients. Age sq = age squared; SACT = Sustained-Attention-to-Cue-Task.

Table S10

Partial regression coefficients for testing moderating effects of age on the association between WCST and emotional granularity

Variable	Negative emotional granularity			Positive emotional granularity		
	B	*p* (two-tailed)	β	*B*	*p* (two-tailed)	β
			Sample 1			
Intercept	–1.224	–	–	–1.286	–	–
Age	0.054	.019	.219	0.005	.756	.029
Age sq	–0.005	.700	–.031	0.015	.138	.120
WCST	–0.011	.227	–.204	–0.009	.169	–.233
WCST x Age	–0.006	.196	–.160	–0.002	.566	–.071
WCST x Age sq	0.004	.098	.159	0.003	.091	.162
			Sample 2			
Intercept	–1.335	–	–	–1.404	–	–
Age	0.034	.046	.152	–0.020	.143	–.195
Age sq	0.012	.256	.084	0.021	.012	.190
WCST	0.009	.012	.235	0.001	.739	.034
WCST x Age	0.003	.211	.113	–0.001	.590	–.049
WCST x Age sq	–0.002	.131	–.112	0.000	.682	.032
			Combined sample			
Intercept	–1.288	–	–	–1.353	–	–
Age	0.041	.003	.174	–0.009	.376	–.052
Age sq	0.005	.558	.032	0.018	.005	.154
WCST	0.006	.059	.154	–0.001	.766	–.024
WCST x Age	0.002	.398	.058	–0.000	.821	–.016
WCST x Age sq	–0.001	.486	–.039	0.001	.212	.070

Note. Age in years was divided by 10 (such that one unit corresponds to one decade) in order to increase regression coefficients. Age sq = age squared; WCST = Wisconsin Card Sorting Test.

Table S11

Partial regression coefficients for testing moderating effects of age on the association between FIST and emotional granularity

Variable	Negative emotional granularity			Positive emotional granularity		
	B	*p* (two-tailed)	β	*B*	*p* (two-tailed)	β
			Sample 1			
Intercept	–1.233	–	–	–1.284	–	–
Age	0.056	.041	.223	0.004	.858	.019
Age sq	–0.012	.459	–.073	0.024	.043	.197
FIST	0.001	.675	.048	–0.03	.031	–.246
FIST x Age	–0.001	.439	–.085	0.001	.154	.155
FIST x Age sq	0.000	.789	.023	0.000	.153	.122
			Sample 2			
Intercept	–1.325	–	–	–1.391	–	–
Age	0.034	.073	.155	–0.016	.269	–.095
Age sq	0.010	.342	.073	0.024	.004	.221
FIST	–0.002	.201	–.133	–0.002	.269	–.158
FIST x Age	0.000	.954	.005	0.001	.462	.067
FIST x Age sq	0.000	.383	.068	0.001	.197	.100
			Combined sample			
Intercept	–1.288	–	–	–1.345	–	–
Age	0.045	.004	.190	–0.011	.348	–.062
Age sq	0.001	.909	.007	0.022	.001	.192
FIST	–0.001	.512	–.050	–0.003	.010	–.197
FIST x Age	–0.000	.601	–.036	0.001	.156	.097
FIST x Age sq	0.000	.366	.050	0.001	.084	.096

Note. Age in years was divided by 10 (such that one unit corresponds to one decade) in order to increase regression coefficients. Age sq = age squared; FIST = Flexible Item Selection Task.

5. Exploratory results for regressing negative and positive emotional granularity indices on all executive function task scores simultaneously

Table S12

Partial regression coefficients for simultaneously regressing negative and positive emotional granularity on executive function task scores in the two samples and the combined sample

Variable	Negative emotional granularity			Positive emotional granularity		
	B	*p* (one-tailed)	β	*B*	*p* (one-tailed)	β
			Sample 1			
Intercept	–1.484	–	–	–0.416	–	–
VCST	–0.000	.516	–.004	–0.002	.774	–.075
SNT	–0.003	.674	–.051	–0.001	.578	–.022
AST	0.001	.284	.058	–0.002	.856	–.106
SACT	0.002	.215	.080	–0.000	.501	–.000
WCST	0.003	.368	.033	–0.005	.770	–.070
FIST	0.000	.524	–.007	–0.001	.644	–.041
			Sample 2			
Intercept	–1.325	–	–	–1.664	–	–
VCST	–0.001	.570	–0.014	0.002	.190	0.073
SNT	–0.004	.816	–0.074	0.000	.525	–0.004
AST	0.002	.146	0.090	0.003	.025[a]	0.171
SACT	0.001	.310	0.040	–0.002	.820	–0.077
WCST	0.005	.052	0.126	0.002	.245	0.053
FIST	–0.004	.993	–0.227	–0.002	.901	–0.116
			Combined sample			
Intercept	–1.479	–	–	–1.352	–	–
VCST	0.000	.555	–.009	0.001	.351	.025
SNT	–0.003	.786	–.052	–0.001	.634	–.023
AST	0.002	.139	.070	0.001	.163	.063
SACT	0.002	.154	.063	–0.001	.766	–.045
WCST	0.005	.071	.087	0.002	.237	.043
FIST	–0.003	.974	–.136	–0.001	.925	–.101

Note. VCST = Visual-Verbal Complex Span Task; SNT = Spatial *n*-Back Task; AST = Antisaccade Task; SACT = Sustained Attention-to-Cue Task; WCST = Wisconsin Card Sorting Test; FIST = Flexible Item Selection Task.
[a] *p*-value not under critical threshold after false discovery rate correction.

6. Correlations between specific error scores of executive function tasks and emotional granularity indices

Table S13

Correlations of specific error scores of executive function task scores and emotional granularity indices in Samples 1 and 2 and the combined sample

Variable	Sample 1		Sample 2		Combined sample	
	r	*p* (one-tailed)	*r*	*p* (one-tailed)	*r*	*p* (one-tailed)
Negative emotional granularity						
SNT omissions	–.01	.449	.13	.971	.07	.893
SNT false alarms	.01	.567	–.05	.216	–.02	.362
WCST drift errors	–.05	.286	–.05	.237	–.06	.148
WCST persevera-tion errors	–.05	.271	–.08	.126	–.08	.076
Positive emotional granularity						
SNT omissions	.08	.849	–.01	.432	.02	.676
SNT false alarms	.10	.881	–.03	.354	.04	.755
WCST drift errors	–.10	.118	.01	.581	–.03	.277
WCST persevera-tion errors	.01	.534	–.08	.113	–.06	.111

Note. Significance of the correlations was evaluated with one-tailed p-values for expected negative correlations (given that higher error scores reflect poorer performance). SNT = Spatial *n*-Back Task; Wisconsin Card Sorting Test.

7. Relations of executive functions with between-category and within-category indices of negative and positive emotional granularity

Our 15 negative and 12 positive emotion items represented five negative higher-order emotion categories (i.e., anger, fear, sadness, shame, and hopelessness) and four positive higher-order emotion categories (i.e., joy, interest, love, and satisfaction), with each category represented by three items. In addition to indices of overall negative and positive emotional granularity (i.e., integral emotional granularity indices), which were the focus of the manuscript, we also computed indices of negative and positive emotional granularity between categories (i.e., between-category emotional granularity indices) and indices of negative and positive emotional granularity within categories (i.e., within-category emotional granularity indices; Erbas et al., 2019). This allowed us to explore whether the results of our main analyses (based on the integral emotional granularity indices) also hold for emotional granularity indices that account for the level of specificity at which individuals differentiate between emotions.

To compute the negative and positive between-category emotional granularity indices, we first aggregated the items representing the same categories. We then computed the intraclass correlation coefficients (ICC[3,*k*]) over these means for negative and positive emotions separately. We excluded negative ICCs because they cannot be meaningfully interpreted (Erbas et al., 2019). To compute the negative and positive within-category emotional granularity indices, we first computed ICCs(3, *k*) over the items within each category, with negative values subsequently removed from analyses. We then took the means of the category-specific ICCs across categories to obtain aggregated within-category indices of negative and positive emotional granularity. We only computed aggregated scores for participants who had at least two non-negative category-specific ICCs for each valence. We Fisher *Z*-transformed and inverted all indices so that higher values (i.e., values closer to zero) indicated higher between-category or within-category emotional granularity.

Table S14 presents the correlations between the executive function task scores and the between-category and within-category emotional granularity indices for Samples 1 and 2, Table S15 for the combined sample. Note that we report one-tailed *p* values for comparability with the main analyses. We corrected for multiple testing by applying Benjamini and Hochberg's (1995) procedure for controlling for the false discovery rate (FDR) across the 12 significance tests (6 executive function tasks x 2 types of indices) of the correlations for each valence separately. None of the correlations were significantly greater than zero after FDR correction.

Next, we describe the correlations between the latent executive function factor and the four specific emotional granularity indices. We corrected for multiple testing by applying Benjamini and

Hochberg's (1995) procedure for controlling the FDR across the two significance tests (1 executive function factor x 2 types of indices) for each valence separately.

In Sample 1, the common executive function factor was not significantly positively correlated with either of the four specific emotional granularity indices (negative between-category emotional granularity index: r = .05, z = 0.60, one-tailed p = .300; negative within-category emotional granularity index: r = –.09, z = –0.99, one-tailed p = .838; positive between-category emotional granularity index: r = –.06, z = –0.64, one-tailed p = .738; positive within-category emotional granularity index: r = –.24, z = –2.39, one-tailed p = .992).

In Sample 2, the common executive function factor was not significantly positively related to three of the four specific emotional granularity indices (negative between-category emotional granularity index: r = –.01, z = –0.16, one-tailed p = .561; negative within-category emotional granularity index: r = –.14, z = –1.84, one-tailed p = .967; positive within-category emotional granularity index: r = –.05, z = –0.60, one-tailed p = .726). The p-value of the correlation between the executive function factor and the positive between-category emotional granularity index was < .05, r = .14, z = 1.77, one-tailed p = .039. However, the p-value did not fall below the critical p-value after FDR correction, which is why we do not interpret this correlation as statistically significant.

In the combined sample, the common executive function factor was not significantly positively correlated with either of the four specific emotional granularity indices (negative between-category emotional granularity index: r = .02, z = 0.38, one-tailed p = .353; negative within-category emotional granularity index: r = –.12, z = –1.99, one-tailed p = .977; positive between-category emotional granularity index: r = .06, z = 1.02, one-tailed p = .154; positive within-category emotional granularity index: r = –.13, z = –2.14, one-tailed p = .984). Taken together, the findings of our main analyses that executive functions and emotional granularity were not significantly positively associated also held for (negative/positive) between-category and within-category emotional granularity indices.

Table S14

Descriptive statistics and correlations of executive function task scores and between-category and within-category emotional granularity indices in Samples 1 and 2

Variable	*M*	*SD*	*n*	Correlation with Neg. BC EG		Correlation with Neg. WC EG		Correlation with Pos. BC EG		Correlation with Pos. WC EG	
				r	*p* (one-tailed)	*r*	*p* (one-tailed)	*r*	*p* (one-tailed)	*r*	*p* (one-tailed)
Sample 1											
Neg. BC EG	–0.99	0.40	149	–	–	.70	–	.24	–	.14	–
Neg. WC EG	–0.78	0.30	153	–	–	–	–	.29	–	.34	–
Pos. BC EG	–0.97	0.30	153	–	–	–	–	–	–	.54	–
Pos. WC EG	–0.95	0.26	152	–	–	–	–	–	–	–	–
VCST	83.03	10.30	148	.03	.347	–.06	.854	–.04	.672	–.29	.999
SNT	85.55	7.63	150	.02	.422	–.06	.776	–.05	.725	–.16	.974
AST	79.53	14.44	143	.15	.039[a]	–.08	.835	–.09	.844	–.21	.994
SACT	85.01	15.16	147	.11	.099	.07	.209	.03	.358	–.12	.931
WCST	81.44	7.15	145	.00	.497	.01	.429	.06	.235	–.02	.584
FIST	60.86	21.63	151	.00	.500	–.12	.934	–.10	.885	–.12	.933
Sample 2											
Neg. BC EG	–1.03	0.38	217	–	–	.70	–	.29	–	.18	–
Neg. WC EG	–0.82	0.29	217	–	–	–	–	.27	–	.37	–
Pos. BC EG	–1.06	0.30	218	–	–	–	–	–	–	.58	–
Pos. WC EG	–1.01	0.24	218	–	–	–	–	–	–	–	–
VCST	82.67	10.09	215	–.04	.705	–.11	.946	.09	.097	.01	.423
SNT	85.54	6.76	214	–.02	.608	–.07	.842	.07	.155	–.03	.691
AST	76.48	15.79	206	.04	.262	–.06	.811	.17	.008[a]	.03	.340
SACT	82.93	14.67	207	.05	.254	–.01	.558	.05	.248	–.05	.762
WCST	79.92	9.56	211	.13	.028[a]	.04	.274	.07	.151	.02	.363
FIST	62.34	20.44	218	–.11	.953	–.20	.998	.04	.263	–.08	.870

Note. VCST = Visual-Verbal Complex Span Task; SNT = Spatial *n*-Back Task; AST = Antisaccade Task; SACT = Sustained Attention-to-Cue Task; WCST = Wisconsin Card Sorting Test; FIST = Flexible Item Selection Task; neg. = negative; pos. = positive; BC = between-category; WC = within-category; EG = emotional granularity.

[a] *p*-value not under critical threshold after false discovery rate correction.

Table S15

Descriptive statistics and correlations of executive function task scores and between-category and within-category emotional granularity indices in the combined sample

Variable	*M*	*SD*	*n*	Correlation with Neg. BC EG		Correlation with Neg. WC EG		Correlation with Pos. BC EG		Correlation with Pos. WC EG	
				r	*p* (one-tailed)	*r*	*p* (one-tailed)	*r*	*p* (one-tailed)	*r*	*p* (one-tailed)
Neg. BC EG	–1.01	0.39	366	–	–	.70	–	.28	–	.17	–
Neg. WC EG	–0.80	0.29	370	–	–	–	–	.28	–	.36	–
Pos. BC EG	–1.02	0.30	371	–	–	–	–	–	–	.57	–
Pos. WC EG	–0.98	0.25	370	–	–	–	–	–	–	–	–
VCST	82.82	10.16	363	–.01	.547	–.10	.970	.04	.234	–.11	.985
SNT	85.54	7.12	364	.00	.525	–.07	.995	.02	.377	–.09	.958
AST	77.73	15.30	349	.09	.045[a]	–.06	.877	.08	.062	–.05	.841
SACT	83.79	14.89	354	.08	.078	.03	.305	.05	.178	–.07	.916
WCST	80.54	8.69	356	.09	.043[a]	.04	.232	.08	.067	.02	.352
FIST	61.73	20.92	369	–.07	.897	–.17	.999	–.02	.663	–.10	.972

Note. VCST = Visual-Verbal Complex Span Task; SNT = Spatial *n*-Back Task; AST = Antisaccade Task; SACT = Sustained Attention-to-Cue Task; Wisconsin Card Sorting Test; FIST = Flexible Item Selection Task; neg. = negative; pos. = positive; BC = between-category; WC = within-category; EG = emotional granularity.

[a] *p*-value not under critical threshold after false discovery rate correction.

8. References

Benjamini, Y., & Hochberg, Y. (1995). Controlling the false discovery rate: A practical and powerful approach to multiple testing. *Journal of the Royal Statistical Society: Series B (Methodological)*, *57*(1), 289–300. https://doi.org/10.1111/j.2517-6161.1995.tb02031.x

Chen, F. F. (2007). Sensitivity of goodness of fit indexes to lack of measurement invariance. *Structural Equation Modeling: A Multidisciplinary Journal*, *14*(3), 464–504. https://doi.org/10.1080/10705510701301834

Erbas, Y., Ceulemans, E., Blanke, E. S., Sels, L., Fischer, A., & Kuppens, P. (2019). Emotion differentiation dissected: Between-category, within-category, and integral emotion differentiation, and their relation to well-being. *Cognition and Emotion*, *33*(2), 258–271. https://doi.org/10.1080/02699931.2018.1465894

Miyake, A., & Friedman, N. P. (2012). The nature and organization of individual differences in executive functions: Four general conclusions. *Current Directions in Psychological Science*, *21*(1), 8–14. https://doi.org/10.1177/0963721411429458

Rosseel, Y. (2012). lavaan: An R package for structural equation modeling. *Journal of Statistical Software*, *48*(2). https://doi.org/10.18637/jss.v048.i02
